# The potential neuroprotective effects of stingless bee honey

**DOI:** 10.3389/fnagi.2022.1048028

**Published:** 2023-02-08

**Authors:** Nurdarina Ausi Zulkifli, Zurina Hassan, Mohd Zulkifli Mustafa, Wan Norlina Wan Azman, Siti Nurma Hanim Hadie, Nurhafizah Ghani, Anani Aila Mat Zin

**Affiliations:** ^1^Department of Pathology, School of Medical Sciences Universiti Sains Malaysia and Hospital Universiti Sains Malaysia, Kubang Kerian, Kelantan, Malaysia; ^2^Centre for Drug Research, Universiti Sains Malaysia, Penang, Malaysia; ^3^Department of Neuroscience, School of Medical Sciences, Universiti Sains Malaysia, Kubang Kerian, Kelantan, Malaysia; ^4^Department of Chemical Pathology, School of Medical Sciences, Universiti Sains Malaysia and Hospital Universiti Sains Malaysia, Kubang Kerian, Kelantan, Malaysia; ^5^Department of Anatomy, School of Medical Sciences, Universiti Sains Malaysia, Kubang Kerian, Kelantan, Malaysia; ^6^Basic and Medical Sciences Unit, School of Dental Sciences, Universiti Sains Malaysia, Kubang Kerian, Kelantan, Malaysia

**Keywords:** stingless bee honey, brain-derived neurotrophic, epileptogenesis, antioxidant, anti-inflammatory, tyrosine receptor kinase B, neuroprotective

## Abstract

Tropical Meliponini bees produce stingless bee honey (SBH). Studies have shown beneficial properties, including antibacterial, bacteriostatic, anti-inflammatory, neurotherapeutic, neuroprotective, wound, and sunburn healing capabilities. High phenolic acid and flavonoid concentrations offer SBH its benefits. SBH can include flavonoids, phenolic acids, ascorbic acid, tocopherol, organic acids, amino acids, and protein, depending on its botanical and geographic origins. Ursolic acid, p-coumaric acid, and gallic acid may diminish apoptotic signals in neuronal cells, such as nuclear morphological alterations and DNA fragmentation. Antioxidant activity minimizes reactive oxygen species (ROS) formation and lowers oxidative stress, inhibiting inflammation by decreasing enzymes generated during inflammation. Flavonoids in honey reduce neuroinflammation by decreasing proinflammatory cytokine and free radical production. Phytochemical components in honey, such as luteolin and phenylalanine, may aid neurological problems. A dietary amino acid, phenylalanine, may improve memory by functioning on brain-derived neurotrophic factor (BDNF) pathways. Neurotrophin BDNF binds to its major receptor, TrkB, and stimulates downstream signaling cascades, which are crucial for neurogenesis and synaptic plasticity. Through BDNF, SBH can stimulate synaptic plasticity and synaptogenesis, promoting learning and memory. Moreover, BDNF contributes to the adult brain’s lasting structural and functional changes during limbic epileptogenesis by acting through the cognate receptor tyrosine receptor kinase B (TrkB). Given the higher antioxidants activity of SBH than the *Apis* sp. honey, it may be more therapeutically helpful. There is minimal research on SBH’s neuroprotective effects, and the related pathways contribute to it is unclear. More research is needed to elucidate the underlying molecular process of SBH on BDNF/TrkB pathways in producing neuroprotective effects.

## Introduction

Several types of honey are well-researched, namely European bee honey (i.e., Manuka honey, jelly bush honey, African jungle honey) and Malaysian bee honey (i.e., Tualang honey and stingless bee honey, SBH; Ahmed and Othman, 2013). Bees are essential in the ecosystem as pollinating and cultivating agents of various plants. It has been used as commercial pollinators, especially stingless bees, due to their non-stinging characteristic (de Oliveira Cruz and de Oliveira Campos, 2009; Bartelli and Nogueira-Ferreira, 2015). Besides, they also play an indispensable role in maintaining genetic variability and fruit quality (Bartelli and Nogueira-Ferreira, 2015). Bee species are divided into *Apis* sp. (honeybee) and *Melipona* sp. (stingless bee). In the 1980s, researchers reported that honey could also be collected from the stingless bee. The study has found more than 600 species, including *Hymenoptera*, *Apidae*, and *Meliponini*, the largest stingless bee group of eusocial bees (Lavinas et al., 2019). These species were classified under 60 genera, and Melipona and Trigona were found to be significant in number (Michener, 2013; Lavinas et al., 2019).

Stingless bees are commonly found in various sub-tropical countries, including Africa, America, Australia, Southeast Asia, and Malaysia (Kek et al., 2014; Vijayakumar and Jeyaraaj, 2014). In Malaysia, more than 30 stingless bee species are from the *Trigona* genus. The most commercialized stingless bee honey is *Geniotrigona thoracica Smith*, *Heterotrigona itama Cockerell*, *Lepidotrigona Terminata Smith*, *Tetragonula fuscobalteata Cameron*, and *Tetraponera Laeviceps* (Kelly et al., 2014). *Heterotrigona itama* is the most popular domesticated stingless bee used in meliponiculture due to its advantages, as it can be easily found, easily kept, and acts as a good pollinating agent for the crop (Kelly et al., 2014; Wong et al., 2019; Shamsudin et al., 2019a). The honey produced by this stingless bee is known as Meliponine honey, SBH, pot-honey, and *Kelulut* honey (in Malaysia; Souza et al., 2006).

Besides honey, stingless bees also produce other products, including propolis, beebread, cerumen, and bee pollen, which have beneficial medicinal properties (Al-Hatamleh et al., 2020). Stingless bees build their nest in hollows of trunks, branches, and tree roots and in ant or termite nests to store their honey collected from nectars, honeydew, and fruit juices (Ávila et al., 2018; Grüter, 2020). In contrast to *Apis mellifera* honeybees, stingless bees stores honey in a little resin pot toward the end of the nest (Kek et al., 2014). Stingless bees have well-defined characteristics, such as vestigial sting, travel shorter distances for food searching, high moisture content, and non-sting morphology (Vazhacharickal, 2016; Ávila et al., 2018; Mokaya et al., 2022). Moreover, stingless bees can collect nectar and honeydew from small flowers due to their smaller body size (2–5 mm) compared to *Apis mellifera* (Vazhacharickal, 2016; Ngaini et al., 2021; Mokaya et al., 2022). *Heterotrigona itama* species can collect more honey than other SBH families, such as *Tetrigona apicalis* and *Tetragonula laeviceps* (Zainin and Hassan, 2021).

SBH has many beneficial effects, including anti-inflammatory, anti-microbial, antibacterial, anti-diabetic, anti-cancer, and wound healing properties (Ávila et al., 2018; Zulkhairi Amin et al., 2018). It was reported that flavonoids and phenolic compounds contribute to the potential beneficial effects *via* antioxidative and anti-inflammatory activities (Cianciosi et al., 2018; Kwon et al., 2019). For example, luteolin, a flavonoid, can reduce seizure scores, glutamate levels, neuronal cell death, and microglial activation in rat hippocampus of the kainic acid (KA)-induced excitotoxicity model (Lin et al., 2016). Therefore, this led to a postulation that SBH has a high potential to combat oxidative stress, a significant cause of neuroinflammation that can result in neuronal apoptosis and death. For instance, high antioxidant content can prevent lipid peroxidation and protective effects on cell membrane integrity *via* catabolism suppression (Sawaya and Marcucci, 2011). Moreover, sub-chronic (35 days) supplementation of SBH improves mice’s spatial working and reference memories. These results might be due to the phenylalanine compound, a dietary amino acid that has memory-enhancing effects by acting directly on BDNF, thus improving brain signaling and physiology (Mustafa et al., 2019).

## Nutritional value and physicochemical properties of SBH

The stingless bee belongs to the *Meliponinae* subfamily of bees and produces honey and other bee products, including pollen, beeswax, propolis, and royal jelly (Rasmussen and Cameron, 2009; Mustafa et al., 2018). In contrast, to sting honey bees, stingless bees store their honey in vertical jars formed of cerumen, containing more flavonoids and antioxidant qualities than other kinds of honey (Vit et al., 2004; Abd Jalil et al., 2017; Alvarez-Suarez et al., 2018). The physical properties of honey samples determine the quality, duration of shelf life, and biological activities. Honey properties and composition depend on climatic and environmental factors (Iglesias et al., 2012). The colour of SBH depends on bee colonies, while the taste depends on the plants of collected nectar (Kulkarni et al., 2017). In comparison to honey bees, SBH has more nutrients and mineral composition. Due to their tiny size, bees may fit within a more significant number of florals, increasing the honey’s polyphenol content. Therefore, it increases the variety of bioactive chemicals found in honey (Kek et al., 2014; Biluca et al., 2016; Ranneh et al., 2018).

Studies have reported that SBH is golden, slightly brown, and translucent (Gomes et al., 2010). Colour intensity represents the concentration of mineral content, phenolic content, and pigments. The presence of colour pigments in honey, such as carotenoids and some flavonoids, is indicated by the absorbance (ABS) ABS_450_ colour intensity (Beretta et al., 2005). A previous study on physicochemical analysis of honey reported higher ABS _450_ values of SBH, indicating that this type of honey contains more colour pigments than other Malaysian honey (Chan et al., 2017). A study demonstrated that the ABS450 values of various kind of Malaysian SBH range between 169.89–805 mAU; while a higher ABS_450_ value in the range of 1029.00–2103.17 mAU were documented in *kelulut* honey (Khalil et al., 2011a; Moniruzzaman et al., 2013b). The dark brown colour intensity indicates the presence of high phenolic content in honey (Ahmed and Othman, 2013). In this context, *kelulut* honey has a high ABS450 value and lower ash content, indicating high mineral content.

Moisture content is one of the honey quality factors since it affects the shelf-life length and microbiological activity stability (Lage et al., 2012; Shahnawaz et al., 2013; Sujanto et al., 2021). Honey’s moisture content changes owing to nectar flux strength, maturity level, beekeeper handling method, extraction condition, processing, and storage method, seasonal weather conditions, and regional humidity of the harvest location (Moniruzzaman et al., 2013a,b; Sujanto et al., 2021). Studies have reported that the moisture content in SBH is higher when compared to *Apis mellifera* honey (Wang and Li, 2011; Silva et al., 2013). Therefore, more outstanding care is needed to handle honey with higher moisture as it can affect the fermentation activity and shelf life (Wang and Li, 2011; da Silva et al., 2013; Silva et al., 2013). To prolong the shelf-life, drying at a low temperature is recommended after harvesting (Chan et al., 2017).

The microbiological stability of honey is determined by its pH since most bacteria prefer neutral or slightly alkaline environments to flourish in (Silva et al., 2009). The organic acid content in honey varies between samples due to the honey’s floral origin, fermentation rate of sugars to alcohol, bee species, and oxidation to carboxylic acids (Mokaya et al., 2022). Honey with a low pH value showed greater microbial stability, thus increasing the shelf-life duration (Lage et al., 2012). Studies have reported that Trigona honey (*Heterotrigona* and *Geniotrigona* spp.) has lower pH and higher free acidity than *Apis mellifera* honey (Shamsudin et al., 2019b). The pH value of Malaysia *kelulut* honey is comparable to the pH value of SBH from other countries. It was reported that the low pH range (3.22–3.69) of *kelulut* can inhibit microorganism growth, thus, preventing contamination of honey (Do Nascimento et al., 2015).

Hydroxymethylfurfural (HMF) content in honey acts as an indicator of honey’s freshness. It is influenced by several variables, including storage conditions, pH level, heating time and temperature, and floral origin (de Almeida-Muradian et al., 2013). HMF is formed due to the reaction between sugars—such as fructose—and acids. To maintain the low HMF level in honey, a proper storage environment with low-temperature exposure plays a significant role (Shapla et al., 2018; Mokaya et al., 2022). A low HMF level indicates fresh and high-quality honey. Meanwhile, high HMF level is associated with degradation, heat exposure, poor handling, and storage conditions. HMF concentration could increase in the heating process caused by the acid-catalyzed dehydration reaction of hexose sugars like fructose and glucose (Singh and Bath, 1997; Gomes et al., 2010; Khalil et al., 2010).

Honey is a concentrated carbohydrate solution composed of various reducing sugars, including fructose and glucose. The sugar content level of SBH differs depending on the geographical origin, the flora, and the vegetation in that region (Biluca et al., 2016; Chuttong et al., 2016; Se et al., 2018). The primary sugar, sucrose, are found in small quantity and will be broken down by a digestive enzyme (Grüter, 2020). It also contains non-reducing sugars such as sucrose and maltose (Ávila et al., 2018; Zulkhairi Amin et al., 2018; Sujanto et al., 2021). A study has demonstrated that the sugar content in SBH ranges between 68% and 73%. Several studies have reported that the reduced sugar content of SBH is more than 60% (Biluca et al., 2016; Chuttong et al., 2016; Se et al., 2018). SBH has a high fructose-reducing sugar level (48.1%) but lower than *Apis mellifera* honey standards (Biluca et al., 2016; Chuttong et al., 2016). A study has demonstrated that the sugar content for *Heterotrigona itama*, *Geniotrigona thoracica,* and *Lepidotrigona terminate* is approximately 35, 34, and 53% of maltose/trehalulose, respectively, (Grüter, 2020). The study has demonstrated that Malaysian *Trigona* SBH has a low concentration level of fructose and glucose (15.4%–24.7%) when compared to *Apis mellifera* (51.0%) (Shamsudin et al., 2019b).

## Phytochemical compounds found in SBH and their potential neuroprotective effects

Phytochemical compounds are essential in determining honey quality due to their potential properties, such as antimicrobial, anticancer, anti-inflammatory, and antioxidant (Mokaya et al., 2022). The concentration and type of phenolic content and total flavonoid content (TFC) for each kind of honey are affected by floral origins, seasonal factors, and species of bee (Saxena et al., 2010; Tenore et al., 2012; Ismail et al., 2016; Ávila et al., 2019). Flavonoid groups found in honey include flavonols (i.e., myricetin, kaempferol, quercetin, isorhamnetin, pinobanksin, rutin, and galangin), flavones (i.e., genkwanin, luteolin, apigenin, tricetin, and chrysin), and flavanones (i.e., pinocembrin and pinostrobin; Hossen et al., 2017; Zulkhairi Amin et al., 2018). A study has reported that the TFC of *Trigona* SBH observed were higher when compared to other Malaysia *Apis* honey (Ismail et al., 2021). Meanwhile, the phenolic group found in honey includes the hydroxybenzoic acids (methyl syringate, gallic acid, syringic acid, benzoic acid, and 4-hydroxybenzoic acid) and hydroxyl-cinnamic acids (chlorogenic, vanillic, caffeic, p-coumaric, and ferulic acids; Hossen et al., 2017; Zulkhairi Amin et al., 2018). A study has reported that the total phenolic content (TPC) of *Trigona* SBH is higher when compared to *Apis Mellifera* (Abu Bakar et al., 2017; Ranneh et al., 2018).

Numerous research has examined the health benefits of polyphenols, including their antioxidant and neuroprotective effects on various disorders related to the nervous system (Table 2; Abd Aziz et al., 2019; Hazirah et al., 2019; Arshad et al., 2020). SBH has a high concentration of polyphenol chemicals, which makes it effective in promoting cell growth, preserving the cellular structure, and protective effect against free radicals in the region of injury (Abd Jalil et al., 2017; Meo et al., 2017; Cianciosi et al., 2018; Mohamad et al., 2018). Gallic, coumaric, caffeic acids, catechin, kaempferol, and apigenin are the primary antioxidants in honey (Chew et al., 2018). Studies have suggested that phenolic substances have protective effects on cell membranes by decreasing lipid peroxidation and removing free radicals (Chaudhuri et al., 2007). A study has claimed that both honey bee honey (HBH) and SBH can counteract reactive oxygen species (ROS; Twilley and Lall, 2014), whereby the phenolic acid extracted from SBH can reduce the production of ROS (Borsato et al., 2014). Kaempferol can inhibit the effects of free radicals, including NO synthase (NOS) and cyclooxygenase-2 (COX-2; Crespo et al., 2008).

Moreover, SBH has also been reported to have many beneficial effects, including anti-inflammatory, anti-microbial, antibacterial, anti-diabetic, anti-cancer, and wound healing properties (Table 3; Ávila et al., 2018; Zulkhairi Amin et al., 2018). SBH showed an outstanding ability to reduce infection, antioxidative and anti-inflammatory activity (Kwapong et al., 2013). The high antioxidant content can prevent lipid peroxidation and has protective effects on cell membrane integrity *via* catabolism suppression (Sawaya and Marcucci, 2011). Studies have demonstrated that flavonoids and phenolic compounds exert potential beneficial effects *via* antioxidative and anti-inflammatory activities (Table 1; Cianciosi et al., 2018; Kwon et al., 2019). For example, luteolin, a flavonoid, can reduce seizure scores, glutamate levels, neuronal cell death, and microglial activation in rat hippocampus of the KA-induced excitotoxicity model (Lin de Sousa, 2016). Therefore, it led to a postulation that SBH has a high potential to combat oxidative stress, a major cause of neuroinflammation that can result in neuronal apoptosis and death. In conclusion, these beneficial properties of SBH due to the compounds might contribute to neuroprotective potentials on various neurological disorders.

**Table 1:** Physicochemical parameters honey samples of various stingless bees species.

Refs	Country of origins	Honey species	pH value	Moisture content	HMF content (mg/kg)	Reducing sugar (g/100 g)	Sucrose (g/100 g)	Properties
Budin et al. (2017); Chan et al. (2017); Al Kafaween et al. (2019); Hazirah et al. (2019); Ranneh et al. (2019); Arshad et al. (2020)	Malaysia	*Trigona*	26.00, 30.42 (g/100 g)					Antioxidant Antibacterial Anti-obesity Anti-inflammatory, Improve memory, Reduces anxiety
Fatima et al. (2018); Mohamad et al. (2018); Nordin et al. (2018)	Malaysia	*Trigona itama*		23.3, 31.7%				Antiproliferative
de Oliveira Neto and Santos (2011); Ávila et al. (2019)	Brazil	*Scaptotrigona bipunctata*	3.97	19.07 (g/100 g)	4.85	60.01	4.65	Antioxidant
Biluca et al. (2020)	Brazil	*Melipona quadriasciata*	6.27, 42.52	3.18, 6.64	52.8, 71.63		0.85, 1.28	Antioxidant Anti-inflammatory
Carvalho et al. (2009); Sousa et al. (2013); Do Nascimento et al. (2015); Biluca et al. (2016, 2020)	Brazil	*Tetragonisca angustula*	3.72, 4.77	23.2, 25.99 (g/100 g)	9.39, 27.99	43, 66.65	0.95, 2.1	Antioxidant Anti-inflammatory
Ng et al. (2017); Fatima et al. (2018)	Malaysia	*Trigona thorasica*		2, 33.7%				Anti-inflammatory
Aziz et al. (2017); Abu Bakar et al. (2017); Selvaraju et al. (2019)		*Geniotrigona thoracica*	3.36	28.17 (g/100 g)	-	-	-	antioxidant
Abu Bakar et al. (2017); Mustafa et al. (2019); Abdul Malik et al. (2020)	Brazil	*Melipona marginata*	2.93, 3.67	32.44, 32.65 (g/100 g)	48.09	63.5, 67.39	0.85	Antioxidant Anti-inflammatory

**Table 2:** List of the main compounds found in SBH and its potential neuroprotective properties.

Compounds	Biological activity (*in vivo/ in vitro*)	Properties	References
Kaempferol	Kaempferol can inhibit oxidative damage and neuroinflammation in spinal cord injury.	Antioxidant, anti-inflammatory	Pan et al. (2020); Ahmed et al. (2021); Liu et al. (2021); Chang et al. (2022); Jafari et al. (2022); Lopez-Sanchez et al. (2022)
	Kaempferol shows neuroprotective effects by reducing neuropathic pain in rats and neuroinflammation in BV2 microglial cells.	Analgesic, anti-inflammatory	
	Kaempferol has neuroprotective effects against Parkinson’s disease model and SH-S5Y5 cells. Kaempferol shows inhibitory effects on oxidative and inflammatory markers.	Antioxidant, anti-inflammatory	
	Kaempferol treatment has neuroprotective against neurodegeneration in the striatum and hippocampus of 3-nitropropionic acid (NPA)-induced model.	Antioxidant, improves memory	
	The study showed that kaempferol attenuates epileptic seizure severity. After kaempferol treatment, the result shows the downregulation of pro-inflammatory and upregulation of anti-inflammatory cytokines in the epileptic model.	Antioxidant, anti-inflammatory	
Catechin	The study showed that catechin attenuates epileptic seizure severity. After catechin treatment, the result shows the downregulation of pro-inflammatory and upregulation of anti-inflammatory cytokines in the epileptic model.	Antioxidant, anti-inflammatory	Ahmed et al. (2021)
Phenylalanine	SBH supplementation has the potential to improve spatial memory performances *via* BDNF signaling	Improves memory, antioxidants	Mustafa et al. (2019)
Chrysin	Chrysin treatment has neuroprotective effects against epileptic rat models by reducing neuronal and oxidative damage.	Antioxidant	Shooshtari et al. (2020); Abd Al Haleem et al. (2021); Zhang et al. (2021)
	Chrysin treatment has neuroprotective effects by attenuating neuronal degeneration, inflammation, and oxidative damage. Besides, also shobehaviordepressant effects by reducing their depressive-like behavior.	Antioxidant, anti-inflammatory, anti-depressant	
	The reported that chrysin has neuroprotective effects by improving cognitive impairments, reduces oxidative damage and induces neurons survival rate.	Antioxidant	
Apigenin	Apigenin has antidepressant effects on chronic stress model by regulating autophagy.	Antidepressant	Zhang X. et al. (2019); Singh et al. (2021); Yadav et al. (2022)
	The study reported that apigenin has neuroprotective effects on neurotoxicity model by inhibiting apoptotic markers, regulating neurotransmitter, downregulate inflammatory markers and reduces oxidative damage.	Antioxidant, anti-inflammatory.	
	Apigenin shows neuroprotective effects on multiple sclerosis-induced rats by reducing pathological changes and demyelination volume. Moreover, restores abnormal apoptotic markers as well as reduces inflammatory cytokine level and oxidative damages.	Anti-apoptotic, anti-inflammatory and antioxidant	
Myricetin	Myricetin has neuroprotective effects on seizure-induced models by regulating molecular pathways involves in epileptogenesis which are BDNF/TrkB, matrix metalloproteinase-9 and GABAA. Besides, it also reduces neuronal damage in hippocampus.	Antioxidant, anti-apoptotic	Sun et al. (2019)
Naringenin	Naringenin has anti-convulsant effects against pentylenetetrazole-induced seizure model.	Anti-convulsant	Copmans et al. (2018); Mokarrami et al. (2022)
	Naringenin has neuroprotective effects against Alzheimer’s model by improving memory performances, and total neuronal numbers.	Antioxidant, improves memory performances	
Caffeic acid	The effects of SBH and caffeic acid have comparable neuroprotective effects against HCHF-induced metabolic syndrome in the rat’s brain	Antioxidant, anti-inflammatory	Arshad et al. (2020); Muhammad Abdul Kadar et al. (2022)
Ferulic acid	Ferulic acid has neuroprotective effects against pentylenetetrazol-induced seizure model by improving learning and memory performances as well as reduces oxidative and neuronal damage.	Improves learning and memory performances,anti-oxidant, anti-apoptotic.	Zhang S. -H. et al. (2019); Park et al. (2022)
	The study reported that ferulic acid has neuroprotective effects against Alzheimer’s disease model by reducing neuroinflammation and oxidative damage.	Anti-inflammatory, antioxidant	
4-hydroxybenzoic acid	SBH has potential protective roles against LPS-induced rat model. SBH supplementation can potentially attenuate inflammation and oxidative stress *via* Nrf2, NF-κB and p38 MAPK signaling	Antioxidant, anti-inflammatory	Ranneh et al. (2019)
Quercetin	The study showed that catechin attenuates epileptic seizure severity. After catechin treatment, the result shows the downregulation of pro-inflammatory and upregulation of anti-inflammatory cytokines in the epileptic model.	Antioxidant, anti-inflammatory	Ahmed et al. (2021)

**Table 3:** List of beneficial properties of different SBH species.

SBH species	Origin	Biological activity (*in vivo/ in vitro*)	Properties	References
*H.itama* sp.	Malaysia	SBH can potentially improve cognitive performance by attenuating anxiety and improving the memory performance of high-carbohydrate high fructose (HCHF). The effects of SBH and caffeic acid have comparable neuroprotective effects against HCHF-induced metabolic syndrome in the rat’s brain	Reduces anxiety, improves memory, Antioxidant, anti-inflammatory	Arshad et al. (2020); Muhammad Abdul Kadar et al. (2022)
	Malaysia	SBH reduces the body weight, BMI, adiposity index and organ weight of a high-fat diet obese rat model. Also, SBH can regulate lipid metabolism and hepatoprotective effects in obese rats.	Hepatoprotective	Mohd Rafie et al. (2018)
	Malaysia	The study reported that SBH promotes the expression level of collagen type I and metalloproteinase (MMP)-1 in the cellular aging of human dermal fibroblast cells.	Antiaging, antioxidant	Abdul Malik et al. (2020)
	Malaysia	The study has reported that SBH inhibits the growth of malignant glioma cells.	Antioxidant, anticancer	Ahmad et al. (2019)
	Malaysia	SBH supplementation has the potential to improve spatial memory performances *via* BDNF signaling	Improves memory, antioxidants	Mustafa et al. (2019)
*G.thoracica sp*	Malaysia	SBH might have potential protective effects on the pancreas against streptozotocin-nicotinamide-induced diabetic rats.	Antidiabetic	Aziz et al. (2017)
*Tetragonula biroi sp*	Indonesia	SBH able to reduce the body weight of streptozotocin-induced diabetic mellitus rats. This might be due to reduction of fats and protein formation.	Antidiabetic	Sahlan et al. (2020)
*Trigona* sp.	Malaysia	SBH has potential protective roles against LPS-induced rat model. SBH supplementation can potentially attenuate inflammation and oxidative stress *via* Nrf2, NF-κB and p38 MAPK signaling	Antioxidant, anti-inflammatory	Ranneh et al. (2019)
	Malaysia	SBH induces the cell viability of the lymphoblastoid cell line (LCL). The high concentration of phenolic and flavonoid compounds presents in SBH has a protective role against oxidative damage.	Antioxidant	Hazirah et al. (2019)
	Malaysia	SBH reduces DNA damages on autism-LCL. This might be due to the antioxidant in SBH thus, prevent oxidative damage.	Antioxidant	Nayan et al. (2020)
	Malaysia	SBH has protective effects against oxidative damage in hydrogen peroxide-induced *in vitro* model and inflammation in LPS-induced *in vitro* model.	Antioxidant, anti-inflammatory	Ooi et al. (2021)
	Malaysia	SBH has potential chemopreventive on colon cancer due to its high antioxidant	Chemopreventive, antioxidant	Saiful Yazan et al. (2016)
	Malaysia	SBH produces significant inhibitory effects by controlling the *Escherichia coli* cell culture growth.	Antimicrobial	Al Kafaween et al. (2019)
	Malaysia	SBH induces proliferation and has beneficial effects on the proliferation of dermal fibroblast cells. Therefore, has potential effects in promoting wound healing.	Wound healing	Nordin et al. (2018)
	Malaysia	Sbh has protective effects on spermatogenesis process in diabetic rats.	Antioxidant	Budin et al. (2017)

## Antioxidant and neuroprotective effects of SBH bioactive compounds

Natural antioxidant compounds in honey, including amino acids, proteins, enzymes, ascorbic acid, carotenoid-like substances, organic acids, Maillard reaction, proteins, and polyphenols, especially flavonoids and phenolic acids, contribute to the various beneficial effects of honey (Zulkhairi Amin et al., 2018). SBH, such as *Geniotrigona thoracica* and *Heterotrigona itama*, have been demonstrated to have more antioxidant activity than Manuka honey (*Apis mellifera*) and higher mineral contents than honey made by Colombian stingless bees (Abu Bakar et al., 2017). Moreover, the higher concentration of polyphenols and phenolic acids contribute to more significant antioxidant effects than other local honey, including Tualang honey (Biluca et al., 2016). *Kelulut* honey contains higher TPC, and higher antioxidant activity was also demonstrated in the ferric-reducing antioxidant potential (FRAP) assay compared to New Manuka honey. Meanwhile, the study also reported higher TFC when compared to Portuguese heather honey and Spanish rosemary honey (Ferreres et al., 1994; Hazirah et al., 2019).

The brain is the most sensitive organ to oxidative stress due to its poor repair capacity, high metabolic rate, and high-level consumption of glucose and oxygen (Halliwell, 1992; Dan Dunn et al., 2015). The brain consumes a ~ 10-fold higher level of oxygen when compared to other organs (Gandhi and Abramov, 2012). Besides, the high polyunsaturated fatty acid in the brain makes it more susceptible to lipid peroxidation. Moreover, the brain is also more vulnerable to hydroxyl radical formation due to the high iron load (Halliwell, 1999; Jellinger, 2003; Mariani et al., 2005). The neurons are more prone to cellular damage caused by excessive free radicals than glial cells (Bolaños et al., 2010). The hippocampus and frontal cortex are the most vulnerable brain regions to damage caused by ROS overproduction (Wang and Michaelis, 2010; Jiang et al., 2017; Roganovic et al., 2019). Moreover, the hippocampus is the most susceptible to neurodegeneration, which is also essential in learning, memory, spatial navigation, and emotional behavior (Rafiee Sardooi et al., 2021).

Oxidative stress plays an essential role in the pathogenesis of various neurological and neurodegenerative diseases, including epilepsy, Alzheimer’s disease, Parkinson’s disease, and amyotrophic lateral sclerosis (ALS; Perry et al., 2002; Migliore et al., 2005; Ashrafi et al., 2007). Overconcentration of oxygen radicals will lead to changes in intracellular calcium ions homeostasis which plays a critical role in neuronal excitability and synaptic transmission. Therefore, it results in the vulnerability of neurons and neuronal loss (Puttachary et al., 2015). The initiating brain lesion and seizure increase ROS production, RNS levels, and oxidative stress (Pearson-Smith and Patel, 2017). The production of free radicals during oxidative stress will cause damage to the structural and functional integrity of neuronal cells in various neurological diseases (Rao et al., 2016; Yaacob et al., 2018). It will cause neuronal insult by protein oxidation, lipid peroxidation and DNA damage (Medda et al., 2020). Neurotoxicity in acute and chronic neurological diseases might be due to oxidative stress due to excessive activation of glutamate receptors (Choi, 1988). Glutamate-induced neurotoxicity led to the overproduction of ROS and RNS, resulting in damage to lipids, protein, DNA, and neuronal cell death (Lucas and Newhouse, 1957; Dugan et al., 1995).

The high oxidative stress will cause a neurotoxic effect which is also a critical factor in cognitive dysfunctionalities, including learning and memory performances (Özbalci et al., 2013; Engwa, 2018). The study has shown a significant correlation between antioxidant activity and polyphenols concentration (Kamaruzzaman et al., 2019; Mohamed et al., 2012). Phenolic compounds in honey can inhibit ROS formation as a defence mechanism against oxidative stress (Mijanur Rahman et al., 2014). In an *in vitro* model using primary hippocampal neuronal cells, apigenin, a phenolic substance, exerts a protective effect against harm brought on by oxygen–glucose deprivation and reperfusion (Han et al., 2012). Honey contains p-coumaric acid, kaempferol, luteolin, and apigenin, reducing potential oxidative stress (Ferguson et al., 2005). Apigenin prevents KA-induced excitotoxicity in the hippocampus in a dose-dependent manner by quenching reactive oxygen species and preventing glutathione (GSH) depletion (Han et al., 2012). This compound has demonstrated ROS scavenging activity and prevents the GSH level reduction in the KA-induced excitotoxicity model (Han et al., 2012). Other compounds, such as caffeic acid and catechin, were reported to play an essential role in learning and memory (Sutherland et al., 2006; Hu et al., 2008). Antioxidants exert protective effects by attenuating ROS and thus inhibiting unwanted chain reactions (Ha et al., 2019).

SBH extract can reduce the production of ROS, leukocyte migration, and oedema, contributing to anti-inflammatory impact (Borsato et al., 2014). Both *in vivo* and *in vitro* studies have shown that certain flavonoids significantly stabilize the lysosomal membrane (Leelaprakash and Dass, 2011). Studies conducted *in vitro* have demonstrated that phenolic compounds can exert antioxidant activity by acting on lipid bilayers, particularly those found in lipoprotein domains and cell membranes, which are lipid peroxidation targets (Chaudhuri et al., 2007). Lysosomal stabilization is essential for managing the inflammatory response because it prevents the extracellular release of lysosomal components of activated neutrophils, such as bacterial enzymes and proteases, which can worsen tissue inflammation and damage. Inflammation can cause the release of lysosomal enzymes, leading to several disorders (Leelaprakash and Dass, 2011).

Studies have demonstrated that SBH significantly ameliorates oxidative stress through nuclear factor kappa-light-chain-enhancer (NF-κB) p65 and p38 mitogen-activated protein kinase (MAPK), and Nrf2 expression in lipopolysaccharide LPS-induced rats (Ranneh et al., 2019). Moreover, the antioxidant activity of SBH was demonstrated against LPS-induced inflammation by inhibiting nitric oxide production *via* nitric oxide synthase (Ooi et al., 2021). Studies have proven that consuming honey can decrease oxidative damage, enhance cognitive performance, and suppress memory and learning decline. Antioxidants in honey play a vital role in suppressing anxiety-like behavior in mice (Barros et al., 2006). *Kelulut* honey, can ameliorate anxiety behavior, significant memory retention and protective effects on pyramidal neuronal cells in the hippocampus brain region (Arshad et al., 2020). Besides, honey also showed positive neuroprotective effects on lead-induced neurotoxicity based on open field test and Morris water maze results as well as significant increase in antioxidant activities (catalase, SOD and GSH) results (Abdulmajeed et al., 2016).

## Anti-neuroinflammatory effects and SBH bioactive compounds

Brain inflammation due to brain disruption triggers inflammatory factors secretion from neurons, astrocytes, and microglia, including interleukin-1*β* (IL-1β) and interleukin-6 (IL-6), interferon-*γ* (IFNγ), tumor necrosis factor-*α* (TNF-α) and transforming growth factor-β (TGF-β) (Vezzani et al., 2011, 2013; Kim et al., 2012). Inflammation in the brain causes the disruption of synaptic signaling and plasticity, impairs inhibitory transmission and blood brain barrier (BBB) permeability, lower the seizure threshold and promotes epileptogenesis (Abbott, 2000; Weissberg et al., 2011; Vezzani et al., 2013). Neuroinflammation involves the generation of seizures and directly affects neurovascular and glial functions (Sternberg, 2000; Vezzani et al., 2011, 2012). Meanwhile, systemic inflammation triggers epileptiform neuronal discharge through ionic and neurotransmitter physiology, which are the consequences of impairments in the blood–brain barrier (Sternberg, 2000; David et al., 2009; Heinemann et al., 2012).

SBH antioxidant effect prevents the signaling of NF-ĸB and MAPK also reduces the production of inflammatory factors IL-6, TNF-α and IFN-γ (Ranneh et al., 2019; Biluca et al., 2020). Flavonoid compounds such as quercetin act as anti-inflammatory and antioxidant agents. Several studies have shown that SBH can reduce proinflammatory cytokines IL-6, interleukin-2p70 (IL-2p70), TNF-α and IFN-γ and anti-inflammatory cytokine, IL-10 expression levels. These substances can inhibit the generation of iNOS, COX-2, cytokine production, nicotinamide adenine dinucleotide phosphate (NADPH) oxidase, ROS, and proinflammatory mediators (Vezzani et al., 1999; Wu et al., 2016). Flavonoids can directly affect cellular receptors and proteins (kinases and enzymes), which can cause physiological changes and changes in the way genes are expressed. This could help protect neurons by making inflammatory responses more effective, which can directly change neuroinflammatory cascades or get rid of free radicals (Copmans et al., 2018).

High antioxidant substances in honey are used in studies related to inflammation and the associated diseases, as oxidative stress is the critical factor in determining the inflammatory status (Arulselvan et al., 2016; Al-Hatamleh et al., 2020). Antioxidant activity prevents inflammation from getting worse by blocking enzymes from working. These enzymes are nitric oxide synthases (NOS), cytochrome P450 (CP450), NADPH oxidase (NOX), xanthine oxidase (XO), lipoxygenase (LO), cyclooxygenase (COX), and myeloperoxidase (MP). The latter prevents ROS production, reducing oxidative stress and producing an anti-inflammatory effect (Al-Hatamleh et al., 2020). Flavonoids inhibit enzymes and other inflammatory mediators such as reactive C protein or adhesion molecules. One strategy to alter the sequence of molecular occurrences that leads to the overexpression of those mediators is to suppress transcription factors like NF-ĸB and activator protein-1 (AP-1) by inhibiting protein kinases involved in signal transduction. The study has reported that flavonoids potentially reduce inflammation by boosting antioxidant defences by activating the NF-E2-related factor 2 (Nrf2) (Leelaprakash and Dass, 2011; González-Gallego et al., 2014).

Several studies have demonstrated the ability of SBH to exert various beneficial effects due to the high concentration and diversity of phenolic acid and flavonoid compounds (Sousa de Sousa, 2016; de Oliveira et al., 2017; Alvarez-Suarez et al., 2018; Souza et al., 2021). For example, antioxidant and anti-inflammatory effects due to flavonoids and polyphenols content improve cognitive disabilities and protective effects *via* neuronal and glial pathways (Mohamed et al., 2012; Kamaruzzaman et al., 2019). Studies have also shown that ursolic acid, p-coumaric acid, and gallic acid might lessen apoptotic symptoms in neuronal cells, such as nuclear morphological alterations, DNA fragmentation, and cell blebbing (Hong et al., 2012). Flavonoids such as quercetin, resveratrol, apigenin, kaempferol, epicatechin, and curcumin can influence mitochondrial processes. These compounds act by inhibiting enzymes, scavenging ROS, or altering the activity of different transcription factors involved in oxidative pathways (Gao et al., 2007; Lagoa et al., 2011; Gibellini et al., 2015).

## Cognitive, learning, and memory function and SBH bioactive compounds

Several studies have demonstrated the abilities of honey to promote cognitive functions in the inflammatory-induced model due to the presence of various compounds (Arshad et al., 2020; Yaacob et al., 2020; Adeniyi et al., 2022) Honey can improves anxiety, cognitive behavioral and locomotor activity dysfunctionalities in lipopolysaccharide-induced neuroinflammation model (Adeniyi et al., 2022). SBH and its phenolic content prevent neuroinflammation, act against neuronal damage caused by neurotoxins, and induce memory, learning, and cognitive function in the brain (Rao et al., 2016; Meo et al., 2017; Yaacob et al., 2018). Moreover, the high antioxidant activity of quercetin, a flavonoid compound in SBH, promotes memory performance and synaptic plasticity of hippocampal neuronal cells in the chronic lead exposure experimental model (Mijanur Rahman et al., 2014). *Kelulut* honey can improve spatial memory impairment in the LPS-induced rats model and anxiety in the metabolic syndrome rat model (Arshad et al., 2020; Yaacob et al., 2020).

Previously, studies have shown that caffeic acid (phenolic acid) and catechin (flavonoids) play an essential role in learning and memory performances (Sutherland et al., 2006; Hu et al., 2008). Other bioactive compounds, including kaempferol, catechin, quercetin, and caffeic acid found, which are also present in SBH, have demonstrated protective effects against memory impairment in various (Dajas et al., 2003; Unno et al., 2007; Park et al., 2010; Cai et al., 2011; Anwar et al., 2012; Zulkhairi Amin et al., 2018). Besides, other flavonoid compounds, quercetin, also play a vital role in memory and synaptic plasticity in memory impairment-induced *in vitro* models (Hu et al., 2008; Kumar et al., 2008). The study has reported that honey-supplemented rats showed better memory performances when compared to sucrose-only-fed rats (Chepulis et al., 2009). So, it’s clear that sugar content alone does not improve memory. There are also other compounds in honey that do (Kishore et al., 2011; Khalil et al., 2011a, b).

High flavonoid content in SBH may be comparable to other natural products (green tea, blueberry, and *Ginkgo biloba*) and acts through hippocampus BDNF. Therefore, suggesting that it can contribute to better learning and memory processes (Williams et al., 2008; Li et al., 2009a,b; Hou et al., 2010; Rendeiro et al., 2013). Previously, the flavones compound mimicked neurotrophin’s binding ability by inducing the tyrosine phosphorylation and dimerization of TrkB. Moreover, it can mimic BDNF’s ability to activate the downstream pathways essential in neurogenesis and synaptic plasticity (Jang et al., 2010; Gupta et al., 2013; Wurzelmann et al., 2017; Paul et al., 2021). Furthermore, a study on SBH supplementation showed that it could induce synaptic plasticity and synaptogenesis, thus improving learning and memory performance in the brain (Mustafa et al., 2018). In addition, studies have reported that seizures could stimulate BDNF gene expression levels and upregulate TrkB activation in the epileptic experimental model (McNamara and Scharfman, 2012). Moreover, BDNF contributes to the adult brain’s lasting structural and functional changes during limbic epileptogenesis by acting through the cognate receptor TrkB (Binder et al., 1999; Scharfman et al., 2005).

Studies have demonstrated the amino acid profile and concentration in various honey samples, and the honey’s botanical and geographical origins may affect its phenylalanine content (Shamsudin et al., 2019a). Other study has also highlighted the presence of phenylalanine, a dietary amino acid, in SBH (Uren and Turnley, 2014; Ramli et al., 2019; Slutsky and Etnier, 2019). Previously, studies have suggested a dietary amino acid might have memory-enhancing effects by acting directly on BDNF signaling pathways (Mustafa et al., 2019). This dietary amino acid converts to tyrosine, a vital component of the tyrosine receptor kinase B (TrkB), a BDNF receptor (Lu et al., 2018). Previously, a study has reported that phenylalanine decreases neurite development and promotes neuronal death *via* lowering BDNF mRNA and protein levels (Li et al., 2010). Moreover, accumulated phenylalanine interferes with brain development, causing microcephaly, intellectual incapacity, and behavioral issues. Neuronal cell loss, white matter abnormalities, dendritic simplicity, and synaptic density decrease may cause these problems. However, future research is needed to understand the phenylalanine-BDNF connection (Huttenlocher, 2000; Li et al., 2010).

## SBH as a possible neuroprotective agent and the potential mechanism

SBH consists ten-fold higher concentration of polyphenolic when compared to other honey (Mustafa et al., 2018). Polyphenolic substances including ferulic acid, luteolin, caffeic acid, isoflavones, and flavone can increase the survival, growth, number, and size of neuronal cells (Lin et al., 2010; Yabe et al., 2010; Tangsaengvit et al., 2013; Santos, 2014; Zaidi et al., 2019; Yin et al., 2022). Moreover, other compounds such as genistein, quercetin, liquidity, isorhamnetin, and resveratrol, neurotrophin (nerve growth factor and BDNF) can also help neurites grow (Chen et al., 2009; Nakajima et al., 2011; Xu et al., 2012; Zhang et al., 2012; Karimipour et al., 2019). Other phenolic compounds, like flavonoids (luteolin, daidzein, puerarin, hesperidin) and flavonols (kaempferol, quercetin, and isorhamnetin), can increase the expression of neuron differentiation markers, like neurofilaments light subunit, synaptophysin, and synapsin (Wang et al., 2008; Lin et al., 2010; Xu et al., 2012; Karimipour et al., 2019).

Neurotrophins (NGF, BDNF, NT3, and NT4) and bioactive compounds that activate neurotrophin receptors may be able to treat neurodegenerative and neurological diseases (Xiao and Le, 2016; Machaalani and Chen, 2018). Flavones promote TrkB dimerization and tyrosine phosphorylation to imitate neurotrophin’s binding capacity (Jang et al., 2010; Gupta et al., 2013; Wurzelmann et al., 2017; Paul et al., 2021). In addition, it can stimulate downstream pathways like BDNF. For example, SBH (*H.itama*) enhances spatial memory by acting on BDNF and inositol tri-phosphate (Mustafa et al., 2019). The possible role of SBH works through BDNF and inositol triphosphate to inhibit NF-ĸB and mitogen-activated protein kinase (MAPK), which supressing inflammatory factors (TNF-*α* and IL-6) (Arshad et al., 2020).

### BDNF/TrkB and the associated signaling pathways

**Figure 1:**
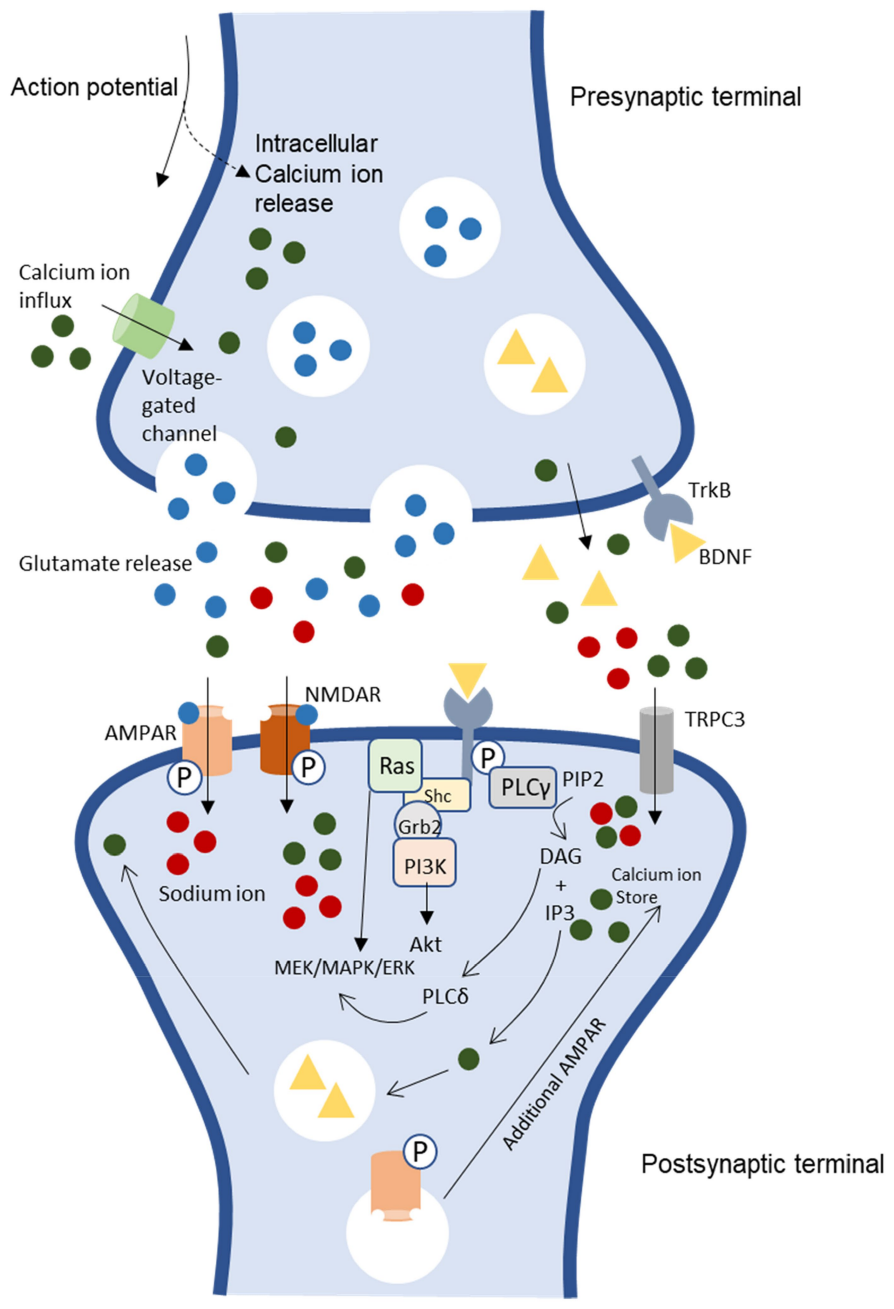
BDNF/TrkB signaling pathways and association to LTP induction and neuronal excitability [CaMKII, Ca^2+^/calmodulin-dependent protein kinase II, NMDAR, N-methyl-D-aspartate receptor, AMPAR. α-amino-3-hydroxy-5-methyl-4-isoxazolepropionic acid receptor, PKC, protein kinase C, TRPC 3, transient receptor potential-3 channels, IP3, inositol 1,4,5-triphosphate, Shc, src homology 2 domain-containing adapter protein, MAPK/ERK, mitogen-activated protein kinase/ extracellular signal-regulated kinases, PLC *γ*, phospholipase C gamma, PI3K, phosphatidylinositol 3-kinase, DAG, diacylglycerol, TrkB, tyrosine receptor kinase B (Murray and Holmes, 2011)].

Neurotrophins are a polypeptide growth factor family that bind to specific tyrosine kinases known as Trk receptors. The dimerization of ligand-initiated receptors and phosphorylation of tyrosine residue in the intracellular Trk domains will start the transduction of the BDNF signal (Segal and Greenberg, 1996; Huang and Reichardt, 2001; Yoshii and Constantine-Paton, 2010). BDNF is synthesized and released in an activity-dependent manner, which will bind specifically to TrkB receptors (Klein et al., 1993; Lu, 2003). The neurotrophin binding to the Trk receptor subsequently activates pathways for development and functioning related to the nervous system (Soppet et al., 1991). Neurotrophin brain-derived neurotrophic factor (BDNF) binds to high affinity to its primary receptor, TrkB. Subsequently, trigger intracellular signaling pathway phospholipase C gamma (PLC *γ*), phosphatidylinositol 3-kinase (PI3K), and mitogen-activated protein kinase/extracellular signal-regulated kinases (MAPK/ERK; Figure 1; Segal and Greenberg, 1996; Park and Poo, 2013).

These downstream signaling pathways are essential in neurogenesis and synaptic plasticity (Tanaka et al., 1997; Mathew and Hablitz, 2008; Yoshii and Constantine-Paton, 2014). ERK is essential for neurogenesis and promotes survival by activating genes that promote survival and suppressing proteins that promote apoptosis (Bonni et al., 1999; Barnabé-Heider and Miller, 2003). Furthermore, MAPK/ERK pathway is essential for regulating protein synthesis-dependent plasticity by upregulating the phosphorylation of ribosomal protein and translation factors (Kelleher et al., 2004; Klann and Dever, 2004). Meanwhile, the TrkB-PI3K-Akt pathway is essential in maintaining synaptic plasticity through protein translation and transportation of synaptic proteins (Sarbassov et al., 2005). This pathway, TrkB-PI3K-Akt acting *via* the mammalian rapamycin target (mTOR), involves protein translation (Sarbassov et al., 2005). Besides, the PI3K-Akt signaling pathway also involves the regulation of synaptic transportation (Yoshii and Constantine-Paton, 2007).

BDNF is essential in trafficking the N-methyl-D-aspartate (NMDA) receptor subunits to the membrane increasing calcium influx and additional BDNF release (Figure 1). The induction of early long-term potentiation, E-LTP, requires both post-synaptic calcium ion (Ca^2+^) influx and activation of the NMDAR complex (Hartmann et al., 2001; Walz et al., 2006). Backpropagating action potentials also influence calcium influx by opening voltage-dependent calcium channels. Sufficient calcium building up locally will lead to postsynaptic BDNF release, thus cause increased presynaptic vesicle cycling. These pathways support synaptic plasticity and LTP (Sabatini et al., 2001; Waters et al., 2005). Moreover, TrkB receptors interact with glutamate receptors due to postsynaptic activation. Once the membrane has sufficiently depolarized, it causes the removal of the magnesium ion (Mg^2+^) block on NMDAR channels, and L-glutamate binding allows NMDAR to open, which triggers Ca^2+^ influx (Bliss and Collingridge, 1993; Lynch, 2004).

Furthermore, BDNF–TrkB triggers cation influx through canonical transient receptor potential-3 channels (TRPC 3). Activating TrkB and PLC depletes IP3-dependent Ca^2+^ stores, triggering Ca^2+^ and sodium ion (Na^+^) influx through TRPC3 and perhaps enhancing synaptic Ca2+ entry *via* voltage-gated channels or NMDARs (Li et al., 1999; Amaral and Pozzo-Miller, 2007). As a result, transient activation of numerous enzymes is necessary to induce E-LTP due to the high spike in intracellular Ca^2+^ concentration and encouragement of Ca^2+^ inflow (Sweatt, 1999). For instance, protein kinase C (PKC) and Ca^2+^/calmodulin-dependent protein kinase II (CaMKII). E-LTP induction also needs the transient activation of CaMKII and PKC, and continuous activation is required to maintain E-LTP (Li et al., 1999; Amaral and Pozzo-Miller, 2007).

CaMKII and PKC become independently active during E-LTP’s maintenance phase (Sweatt, 1999). This causes the phosphorylation of α-amino-3-hydroxy-5-methyl-4-isoxazole propionic acid (AMPA) receptors, enhancing their activity and trafficking to and from the synaptic plasma membrane and insertion of the GluR1 subunit into the postsynaptic membrane (Malenka, 2003; Malenka and Bear, 2004; Shepherd and Huganir, 2007). TrkB and the release of Ca^2+^ from internal IP3-sensitive storage are likely implicated in the mechanisms by which BDNF increased the synaptic delivery of glutamate subunits, GluR1 (Reichardt, 2006; Lin et al., 2009). Studies have reported that LTP depends on AMPA receptor trafficking to the synaptic plasma membrane. Late-long-term potentiation (L-LTP) generated during E-LTP persists for an extended period, up to hours or days, and involves gene transcription and protein synthesis. Due to this, L-LTP induction is a good candidate for long-term molecular memory (LTM; Morris et al., 2003; Sutton and Schuman, 2006; Whitlock et al., 2006; Zhou et al., 2006).

### BDNF/TrkB signaling association in synaptic plasticity

In the hippocampus, LTP is characterized by an activity-dependent change in synaptic strength and cellular processes that underlie learning and memory (Bliss and Collingridge, 1993). Various studies have suggested that the synaptic changes triggered by LTP are critical for memory formation and consolidation of long-term memories (Bliss and Collingridge, 1993; Selcher et al., 2002). Modifications in the individual excitatory synaptic strength promote the refinement and information storage related to learning and memory. E-LTP will elevate the strength of synapses within a short period, while higher stimulation will last for a longer duration, also known as L-LTP (Huang and Kandel, 1994). Besides LTP, LTD is a type of activity-dependent synaptic weakening that lasts long. LTD is crucial in synaptic connectivity refinement throughout development (Dudek and Bear, 1993). LTD or depotentiation mechanisms might decrease hyperexcitability due to the similarities of low-frequency stimulation patterns with LTD induction (Albensi et al., 2004).

Studies have demonstrated a high correlation between hippocampus neurogenesis and cognitive ability (Zhou et al., 2004). Improvement of learning and memory abilities are associated with increased neurogenesis and new neurons formed in the hippocampus during enrichment, which is critical for improving the ability to store new information in long-term memory (Bruel-Jungerman et al., 2005). BDNF–TrkB signaling action on the postsynaptic neuron is crucial for excitatory synaptic transmission and LTP induction (Yury et al., 2002). An increase in neuronal excitability will induce synaptic plasticity thus, enhance BDNF signaling. A study has suggested that BDNF–TrkB signaling triggers glutamate release and binds to postsynaptic receptors for LTP induction. The reduction or absence of BDNF and TrkB cause LTP inhibition and learning dysfunctionality (Korte et al., 1995; Figurov et al., 1996; Minichiello et al., 1999). Therefore, shows that BDNF is crucial in neurogenesis, axonal and dendritic sprouting and LTP *via* a TrkB-dependent manner (Patapoutian and Reichardt, 2001). However, this might cause neuronal hyperactivity, resulting in excitotoxicity (Patapoutian and Reichardt, 2001).

Studies have reported that NMDAR subunits, NR2A-NMDA receptor plays an essential role in long-term potential (LTP) production, and the NR2B-NMDA receptor plays a vital role in long-term depression (LTD) induction in the CA1 region of hippocampal brain region (Liu et al., 2004; Evans, 2008). A study has shown that the expression level of NMDA subunit receptor levels changes upon the application of BDNF. The expression level of NR1 and NR2A increase while NR2B decreases (Glazner and Mattson, 2000). Calcium ions released upon activation of NMDA receptors can cause necrosis (cell swelling) and decrease apoptosis (cell shrinkage and chromatin fragmentation; Farmer et al., 2004). These properties might be beneficial and harmful because they induce hyperexcitability in the dentate gyrus neuronal circuit, resulting in widespread excitotoxicity (Scharfman et al., 1999; Feng et al., 2003; Koyama et al., 2004). Dysfunctionality in excitatory neuronal networks might play a pivotal role in various diseases (Bliss and Collingridge, 1993). NMDA and AMPA receptors are involved in the induction of LTP and epileptogenesis (Liu et al., 2004; Evans, 2008).

BDNF release and Trk receptor activity trigger the development of hyperexcitable circuits, and synaptic plasticity and promote NMDA receptors in the exercise KA model (Koyama et al., 2004). The application of BDNF activates the sodium channel and triggers the influx of calcium ions. Therefore, resulting in postsynaptic neuron depolarization of dentate granule cells (Kafitz et al., 1999). High stimulation will trigger calcium ion influx *via* NMDA receptors activation, and AMPA receptors generate excitatory postsynaptic potentials, EPSC (Nowak et al., 1984; Malenka et al., 1988; Nicoll and Malenka, 1999). Both AMPA/ kainate receptor-mediated EPSCs are involved in moderating fast glutamatergic synaptic activity (Represa et al., 1987; Cossart et al., 2002). Besides, BDNF also facilitates excitatory synaptic transmission in the hippocampal CA1 region (Kang and Schuman, 1995). However, the excessive calcium ion influx can lead to glutamate excitotoxicity *via* NMDA receptors, which contribute to brain insults such as anoxia, ischemia, and seizure, thus resulting in neuronal damage (Meldrum, 1993; Murray and Holmes, 2011).

### BDNF/TrkB signaling association in neurological disorder

Activation of the TrkB receptor also plays a pivotal role in the central nervous system in promoting excitatory glutamate-mediated synaptic transmission and inhibitory synaptic transmission. BDNF and its receptor, TrkB, are highly expressed in neuronal and astrocytes in several brain regions, including the hippocampus and cerebral cortex (Croll et al., 1999; He et al., 2004; Holt et al., 2019). Various studies have shown that BDNF plays a vital role in exerting neuroprotective effects against glutamate toxicity (Almeida et al., 2005; Jiang et al., 2005). The pattern of BDNF expression observed in this study was similar to the pattern shown in various neurodegenerative models. BDNF as a treatment application significantly reduces neuronal cell death in the neurotoxin-induced model *via* ERK-induced signaling pathways (Ventimiglia et al., 1995; Bogush et al., 2007). Therefore, it shows that BDNF might exert neuroprotective effects against neuronal insults.

Studies on the relationship mechanism between neuronal plasticity and seizures were usually associated with the hippocampus brain region (Chen et al., 2016). Studies have suggested that the elevated neurotrophic factors expression level following seizure induction might be associated with maintaining long-lasting structural and functional changes contributing to epileptogenesis (Merlio et al., 1993; Conner et al., 1997). BDNF is crucial in developing and maintaining neuronal populations within the central nervous system BDNF can be found in neurons, astrocytes, and microglia, which have a pivotal role in neuronal excitability in various conditions, including epilepsy (Parpura and Zorec, 2010). BDNF expression level was upregulated in pyramidal neuronal cells in the hippocampal region, amygdala, entorhinal cortex, and piriform cortex (Conner et al., 1997). The principal neurons in the hippocampus are excitatory neurons that utilize glutamate receptors to generate fast-acting postsynaptic currents (EPSCs; Yehezkel, 2008). These brain regions are the main areas affected during epilepsy (Binder et al., 2001).

The expression level of BDNF changes during neurotoxicity and in neurodegenerative diseases (Ballarín et al., 1991; Tanila, 2017). Neurotoxin-induced neurodegenerative model induces robust upregulation of BDNF expression levels (Ballarín et al., 1991; Sathanoori et al., 2004). The upregulation of BDNF expression level induces glutamatergic transmission, and limbic circuit neural activity cause potentiation induction (Patterson et al., 1992). BDNF also triggers axonal growth, promotes basal dendritic granule cells formation, facilitates dendritic spine morphological changes, and triggers neurogenesis in the adult brain, such as the dentate gyrus (Lowenstein and Arsenault, 1996; Murphy et al., 1998; Wilson Horch et al., 1999; Danzer et al., 2002; Lee et al., 2002; Tyler and Pozzo-Miller, 2003). The study suggested that the growth-associated activity induced by BDNF might contribute to excitability changes, epileptogenesis, and chronic epilepsy (Lähteinen et al., 2004).

Robust upregulation of BDNF expression was observed after seizures regardless of the method applied (Bengzon et al., 1993; Humpel et al., 1993). Activity-dependent BDNF release and Trk receptor activity cause abnormal mossy fiber sprouting resulting in hyperexcitable circuits activity (Koyama et al., 2004). The study has shown that BDNF does not increase severity in the KA-induced model but the severity of injury in pyramidal neurons of the CA3 hippocampal region. Brain insult, hypoxia, and seizure cause the expression level of BDNF mRNA level to increase in dentate granule cells, promoting BDNF release from mossy fiber and anterograde transport. Therefore, it resulted in the activation of TrKB receptors in hilus and CA3 stratum lucidum. The study explains that vital excitatory mossy fiber input into CA3 pyramidal cells might primarily promote epileptogenesis through BDNF (Binder et al., 2001).

## Conclusion

The bioactive compounds in SBH contribute to antioxidant and anti-inflammatory effects, which is crucial to combat various neurological disorders. These beneficial effects might contribute to the neuroprotective effects by preventing further damage to the central nervous system. Studies have suggested that flavonoids and phenolic compounds present in honey might potentially reduce or prevent these behavioral and cognitive abnormalities caused by oxidative stress, protein processing, neurotrophic signaling, synaptic dysfunction, inflammation, and cell death. The flavones compound can mimic the ability of BDNF on the downstream pathways that is essential in neurogenesis and synaptic plasticity. Moreover, before considering phenylalanine compounds as treatments for various neurological-related disorders, more studies are required. Studies are needed to learn more about the effects of neurotrophic factors and how they work at the molecular level. These results could help build better treatments for neurological disorders and dysfunctions. More investigation is required to fully understand the bioactive substances, molecular processes, and essential elements of SBH that influence the nootropic activity to build this new potential quality standard. This entails developing new delivery methods, such as nanoparticles or other formulations that promote brain uptake, then assessed in the same model to determine the most effective.

## Author contributions

All authors listed have made a substantial, direct, and intellectual contribution to the work and approved it for publication.

## Funding

This article is funded by the Ministry of Higher Education Malaysia for Fundamental Research Grant Scheme with Project Code: FRGS/1/2021/SKK06/USM/02/13 and Universiti Sains Malaysia RU Top-down 2020; 1001/PPSP/8070015.

## Conflict of interest

The reviewer SLT declared a past co-authorship with the author SNHH to the handling editor. The authors declare that the research was conducted in the absence of any commercial or financial relationships that could be construed as a potential conflict of interest.

## Publisher’s note

All claims expressed in this article are solely those of the authors and do not necessarily represent those of their affiliated organizations, or those of the publisher, the editors and the reviewers. Any product that may be evaluated in this article, or claim that may be made by its manufacturer, is not guaranteed or endorsed by the publisher.
